# Effects of uremic solutes on reactive oxygen species *in vitro* model systems as a possibility of support the renal function management

**DOI:** 10.1186/s12882-015-0029-1

**Published:** 2015-04-11

**Authors:** Renata P Assis, Juliana FA Castro, Vânia O Gutierres, Carlos A Arcaro, Renata S Brotto, Olga MMF Oliveira, Amanda M Baviera, Iguatemy L Brunetti

**Affiliations:** Department of Clinical Analysis, School of Pharmaceutical Sciences, São Paulo State University - UNESP, Rua Expedicionários do Brasil 1621, Araraquara, CEP 14801-902 São Paulo Brazil; Department of Biochemistry and Technological Chemistry, Institute of Chemistry, São Paulo State University - UNESP, Rua Prof. Francisco Degni 55, Araraquara, CEP 14800-900 São Paulo Brazil

**Keywords:** Chronic kidney disease, Hemodialysis, Oxidative stress, Uremic solutes, IC_50_ as clinical chemistry tool

## Abstract

**Background:**

In view of the prevalence of oxidative stress in chronic kidney disease (CKD) patients, the loss of low-molecular-weight biomolecules by hemodialysis and the antioxidant potential of some uremic solutes that accumulate in CKD, we used *in vitro* model systems to test the antioxidant potential of the following uremic solutes: uric acid, hippuric acid, *p*-cresol, phenol, methylguanidine, L-arginine, L-tyrosine, creatinine and urea.

**Methods:**

The in vitro antioxidant efficiencies of the uremic solutes, isolated or in mixtures, were tested with the following assays: *i*) ABTS radical cation decolorization assay; *ii*) hypochlorous acid (HOCl/OCl^−^) scavenging activity; *iii*) superoxide anion radical (O_2_^•-^) scavenging activity; *iv*) crocin bleaching assay (capture of peroxyl radical, ROO^•^); *v*) hydrogen peroxide (H_2_O_2_) scavenging activity.

**Results:**

Four of the tested uremic solutes (*p*-cresol, phenol, L-tyrosine, uric acid) were effective antioxidants and their IC_50_ were found in three model systems: ABTS^•+^, HOCl/OCl^−^ and crocin bleaching assay. In the 4-solutes mixtures, each one of the solute captured 12.5% for the IC_50_ of the mixture to ABTS^•+^ or HOCl/OCl^−^, exhibiting a virtually exact additive effect. In the 2-solutes mixtures, for ROO^•^ capture, it was observed the need of more mass of uremic solutes to reach an IC_50_ value that was higher than the projected IC_50_, obtained from the IC_50_ of single solutes (25% of each, in the binary mixtures) in the same assay. In model systems for O_2_^•-^ and H_2_O_2_, none of the uremic solutes showed scavenging activity.

**Conclusions:**

The use of the IC_50_ as an analytical tool to prepare and analyze mixtures allows the determination of their scavenging capacities and may be useful for the assessment of the antioxidant status of biological samples under conditions of altered levels of the endogenous antioxidant network and/or in the employment and monitoring of exogenous antioxidant therapy.

## Background

Oxidative stress has been postulated as a cause and also an exacerbating factor of various diseases, including the chronic kidney diseases (CKD) [1]. Increased reactive oxygen species (ROS) production is often caused by the dysfunctional mitochondria formed in the most important conditions that lead to CKD [2]; impairment in the antioxidant defenses and endogenous activation of phagocytes have been also cited as potential factors responsible for oxidative stress in CKD [3]. Over the last years it has become clear that the association among increased ROS generation, impaired endogenous antioxidant systems and low nitric oxide (NO) bioavailability plays a crucial role in the development of the endothelial dysfunction in CKD patients [4,5], predisposing them to long-term complications closely related to atherosclerosis and cardiovascular morbidity [6,7].

During the progression of the renal disease, loss of kidney function is accompanied by failing organ function leading to accumulation of a series of compounds [8]. So, in the later stages of CKD, the treatment aims to slow the damage progress and to compensate the impairments caused by the reduced kidney function, via a renal replacement therapy, in which hemodialysis (HD) is the most common [2,9]. The primary goal of HD is to restore the intracellular and extracellular fluid environment typical of normal kidney function [10]. However, HD is considered an exacerbating factor for oxidative stress in CKD patients, mainly attributed to the activation of neutrophils during forced passage of blood through the dialysis circuits, provoking endogenous inflammatory processes with release of ROS [11]. Also, loss of circulating low-molecular-weight dialyzable antioxidants is also a consequence of HD. Taken together, it is postulated that oxidative stress increases in CKD patients after HD session [12,13]. Ujhelyi and collaborators [14] found that the decreased antioxidant capacity of plasma ultrafiltrate from CKD patients on HD may be due to the dialytic removal of some uremic solutes, increasing the risk of low density lipoprotein (LDL) oxidation and subsequent endothelial cell damage.

More than 90 uremic solutes removed from blood by HD are reported, grouped according to their physicochemical properties [15]. In spite of the toxicity due to the increased levels of uremic solutes in CKD patients, it is crescent the data regarding the increased oxidative stress after HD attributable to the loss of uremic solutes [16-18], mainly uric acid. Blood levels of O_2_^•-^ are raised in CKD patients and further increased after one session of HD [13]. The controversial roles of uremic solutes in CKD patients highlight to the importance of monitoring their antioxidant status, which can be useful to bring information about the oxidative stress before and after HD, weighting the need of employing antioxidant therapy, as well to investigate the effectiveness of antioxidant interventions.

The assessment in serum of the scavenging capacity against ROS has been cited as a good indicator of the individual defenses to oppose the oxidative stress [19]. The ROS scavenging capacity in biological samples depends on the nature of the reactive species, the molecular structure of the antioxidants and the *locus* of this interaction. However, considering the myriad of molecules acting as antioxidant in serum samples and the interactions among them, it is often difficult to assess the relative contribution of their individual antioxidants capacities [20].

Nowadays, efforts have been made to standardize an in vitro analytical assay attempting to bring information that can expand the actual knowledge of the antioxidant effectiveness in biological samples. In this way, it seems useful to develop analytical tools that allow the determination of the antioxidant capacity of mixtures composed of endogenous and/or exogenous compounds, such as in vitro model systems. Therefore, considering that: *i*) the uremic solutes show different antioxidant capacities and *ii*) the knowledge of their serum levels, we believe that the use of an analytical method that allows their individual scavenging capacity to be determined may be useful for the assessment of the oxidative status in CKD and/or HD patients, as well as for the effectiveness of antioxidant interventions. Data obtained may be useful to correlate with both the prevalence and pathogenesis of cardiovascular diseases and the continuity loss of renal function.

In this light, we investigated the antioxidant activity of the commonest uremic solutes, isolated or in selected mixtures, focusing on the involvement of certain ROS in CKD. To this end, in vitro model systems were used to assess the scavenging activity, with respect to the ABTS cation radical (ABTS^•+^), superoxide anion radical (O_2_^•-^), hypochlorous acid (HOCl), hydrogen peroxide (H2O2) and peroxyl radical (ROO^•^, crocin bleaching assay), exhibited by the following uremic solutes: uric acid, hippuric acid, *p*-cresol, phenol, methylguanidine, L-arginine, L-tyrosine, creatinine and urea.

## Methods

The uremic solutes creatinine, hippuric acid, uric acid, methylguanidine, L-arginine and L-tyrosine were purchased from Sigma-Aldrich, phenol and urea from Merck, and *p*-cresol from Riedel de Haën.

Besides the uremic solutes, 6-hydroxy-2,5,7,8-tetramethylchroman-2-carboxylic acid (Trolox, Sigma Aldrich, USA), a soluble synthetic analogue of vitamin E with well-established antioxidant activity [21], was used as a standard. Absorbance readings were taken in a microplate spectrophotometer (Biotek- Power Wave XS2), or an OceanOptics USB 4000 for the tests with crocin, with magnetic stirring and Peltier heating. The scavenging capacities against oxidizing species were calculated as the mean of triplicate tests, except for peroxyl radical (ROO^•^) scavenging assay, which was performed in duplicate assays. The concentrations reported are final concentrations in the assays.

### ABTS radical cation decolorization assay

Antioxidant activity was assessed by the ABTS method, as described by Re et al. [22], with modifications. The ABTS^•+^ radical cation was generated by oxidation of 2,2′-azino-bis (3-ethylbenzthiazoline-6-sulphonic acid), (ABTS, Sigma Aldrich, USA) (7 mmol/L) with potassium persulfate (Sigma Aldrich, USA) (140 mmol/L) in the dark at room temperature for 12 to 16 hours. This ABTS^•+^ stock solution was diluted in sodium phosphate buffer (10 mmol/L, pH 7.0) to an absorbance of 0.750 ± 0.020, at 734 nm. Various concentrations of uremic solutes were then added, the reaction mixture was incubated for 15 minutes in the dark at room temperature and absorbance was read at 734 nm. The results were expressed as mean ± standard error of the mean (SEM) of the 50% inhibitory concentration (IC_50_).

### Superoxide anion radical (O_2_^•-^) scavenging assay

The O_2_^•-^ was produced by reaction between reduced nicotinamide adenine dinucleotide (NADH, Sigma Aldrich, USA), phenazine methosulfate (PMS, Sigma Aldrich, USA) and molecular oxygen [23]. The O_2_^•-^ generated reacts with nitroblue tetrazolium (NBT, Sigma Aldrich, USA), reducing it to a blue formazan, whose color intensity is directly proportional to the radical concentration. The test was performed in sodium pyrophosphate buffer (25 mmol/L, pH 8.3), containing PMS (372 μmol/L), NBT (600 μmol/L), NADH (1560 μmol/L) and various concentrations of uremic solutes. After 7 minutes at room temperature, the absorbance was read at 560 nm to determine the concentration of formazan [24]. The assay in the absence of uremic solutes was used as a control (100% reaction) and the reaction medium without NADH was used as a reading blank. The results were expressed as mean IC_50_ ± SEM.

### Hypochlorous acid (HOCl/OCl^−^) scavenging assay

The antioxidant activity depends on the capacity of the sample to capture HOCl/OCl^−^, preventing it from oxidizing 3,3′,5,5′- tetramethylbenzidine (TMB, Sigma Aldrich, USA). TMB oxidation by HOCl/OCl^−^ generates a blue compound with maximum absorbance at 655 nm [25,26].

To produce a standard solution of OCl^−^, NaOCl was diluted in 10 mmol/L NaOH and its concentration was determined by its molar extinction coefficient (ε = 350 M^-1^ cm^-1^ at 292 nm) [27].

Various concentrations of uremic solutes in sodium phosphate buffer (50 mmol/L, pH 7.4) were incubated with HOCl/OCl^−^ (30 μmol/L) for 10 minutes. TMB (2.8 mmol/L dissolved in 50% dimethylformamide with 0.01 mol/L potassium iodide in 0.8 mol/L acetic acid) was then added and incubated for 5 minutes at room temperature in the dark and the absorbance were monitored at 655 nm, as described by Dypbukt [26], with modifications. The assay without uremic solutes was used as control (100% reaction) and the absorbance of the reaction medium without HOCl was used as a reading blank. The results were expressed as mean IC_50_ ± SEM.

### Peroxyl radical (ROO^•^) scavenging assay

The peroxyl radical (ROO^•^) is formed during the process of lipoperoxidation (LPO) in aerated medium; several model systems simulate this reaction to assess the capacity of antioxidants to scavenge this type of oxidative activity, including the protection against bleaching of crocin.

The crocin bleaching assay was performed as described by Tubaro et al. [28], by monitoring the decrease in crocin absorbance at 443 nm for 10 minutes, in a competitive kinetics procedure. The reaction is initiated by addition of the azo-compound 2,2′-azobis(2-amidinopropane) dihydrochloride (AAPH, Sigma Aldrich, USA), which by thermolysis at 40°C, generates peroxyl radicals at a constant rate.

**Scheme 1:**
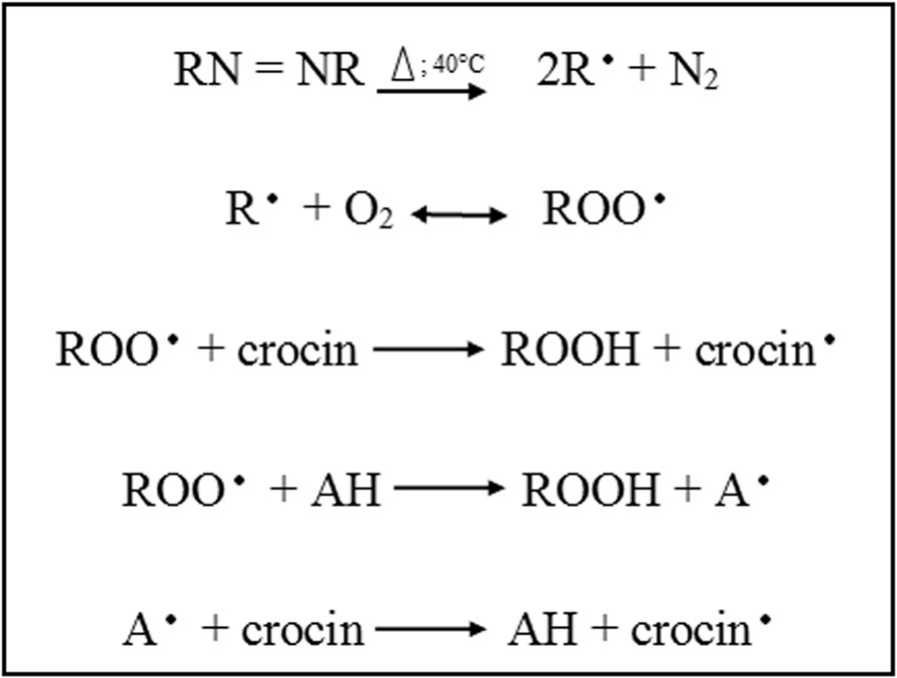
Reactions involved in the crocin bleaching with termolysis of AAPH (adapted from Ordoudi and Tsimidou [58]. A, represents an antioxidant (sample).

Thus, the antioxidants compete with the crocin for ROO^•^; therefore, the inhibition of its oxidation depends on the capacity of samples in capture the radical species (Scheme 1).

To perform this test, the molar extinction coefficient (ε) of crocin in DMSO was determined, as this solvent was used to enhance its solubility and prepare the crocin stock solution: ε = 13,726 M^-1^ cm^-1^, at 443 nm.

The crocin (25 μmol/L, Sigma Aldrich, USA) in sodium phosphate buffer 120 mmol/L, pH 7.0, was mixed with various concentrations of uremic solutes. The reaction was started by adding 12.5 mmol/L of AAPH and performed at 40°C with constant stirring. The rate of crocin bleaching (linear after about 1 minute of reaction) was monitored at 443 nm for 10 minutes. To eliminate possible interference from the sample, a reaction mixture without crocin was prepared for each solute and used as the reaction blank.

The rate of crocin bleaching by the generated peroxyl radical (v_0_) decreases in the presence of an antioxidant, as it competes with the crocin for the peroxyl radical, and the new bleaching rate (v) is given by:

$$ \mathrm{v}={\mathrm{v}}_0\times \frac{\mathrm{kc}\left[\mathrm{C}\right]}{\mathrm{kc}\left[\mathrm{C}\right]+\mathrm{ka}\left[\mathrm{A}\right]} $$

where: v_0_ = k_1_ x [ROO^•^] x [C]; kc = k_1_ x [ROO^•^]; ka = k_2_ x [ROO^•^]; [ROO^•^] = concentration of peroxyl radical; v_0_ = reaction rate between crocin and ROO^•^; k_1_ = rate constant for the ROO^•^-crocin reaction; k_2_ = rate constant for the ROO^•^-antioxidant reaction; [C] = crocin concentration; [A] = antioxidant (uremic solute) concentration.

The fall in crocin bleaching rate in the presence of an antioxidant can be modeled as follows:

$$ \frac{{\mathrm{v}}_0}{\mathrm{v}}=\frac{\mathrm{kc}\left[\mathrm{C}\right]+\mathrm{ka}\left[\mathrm{A}\right]}{\mathrm{kc}\left[\mathrm{C}\right]}=1+\frac{\mathrm{ka}}{\mathrm{kc}}\times \frac{\left[\mathrm{A}\right]}{\left[\mathrm{C}\right]} $$

from the above equation,

The coefficient ka/kc, calculated as the slope of the regression line for the v_0_/v *versus* [A]/[C] plot, indicates the relative capacity of an antioxidant to interact with the peroxyl radicals. By dividing this slope for a uremic solute by the slope for a standard antioxidant such as Trolox, the ratio of rate constants, and thus the relative antioxidant capacity, of the analyzed compound can be estimated, being expressed in Trolox equivalents.

### Hydrogen peroxide scavenging assay

The H_2_O_2_ (Merck, German) oxidizes 2-nitro-5-thiobenzoic acid (TNB, Sigma Aldrich, USA) to 5,5′-dithiobis-2-nitrobenzoic acid (DTNB), with a decrease in absorbance at 412 nm and increase at 325 nm [29].

The TNB solution was prepared by the method of Ching et al. [30]; in 50 mmol/L potassium phosphate buffer (pH 6.6) and its concentration was determined from its molar extinction coefficient at 412 nm (13,600 M^-1^ cm^-1^; [31]); H_2_O_2_ concentration was determined as described by Brestel [32], (ε = 80 M^-1^ cm^-1^, at 230 nm).

In 50 mmol/L potassium phosphate buffer pH 6.6, various concentrations of uremic solutes were incubated with H_2_O_2_ (0.3 mmol/L) for 30 minutes at 37°C. TNB (53 μmol/L) was added and incubated for 1 hour at 37°C. The absorbance was read at 412 nm. Catalase (20 units/mL) was used as a standard H_2_O_2_ scavenging agent.

The percent inhibition of TNB oxidation, i.e., percent H_2_O_2_ capture, was calculated from the difference in absorbance between reaction mixtures with and without uremic solutes.

### Experiments with uremic solute mixtures

In view of the observed effectiveness of some of the uremic solutes, viz*.* uric acid, *p*-cresol, phenol and L-tyrosine, in assays with ABTS^•+^, HOCl/OCl^−^ or ROO^•^, further assays of their ability to scavenge these reactive species were performed on mixtures of these four solutes, in proportions based on their respective IC_50_.

These test mixtures were so designed that each of the 4 (or 2) solutes was present in the same proportion as its IC_50_; thus, fractions or multiples of this volume would correspond to the same fractions or multiples of the IC_50_ of each of the uremic solutes. With this procedure, if the scavenging effect of the solute mixture shows independent activities of solutes, it would be additive and exact, therefore the IC_50_ of the mixture would contain ROS-scavenging activities of each component at 12.5% or 25% for 4 or 2 solutes, respectively, of its individual IC_50_.

## Results

In vitro model systems were used to assay the antioxidant activity against the reactive species: ABTS^•+^, O_2_^•-^, H_2_O_2_, HOCl/OCl^−^ and ROO^•^ (crocin bleaching assay). The assays were performed with uremic solutes at various concentrations, chosen in relation to the upper limit of the respective reference range for healthy people (physiological concentration) and those found in the serum of patients with CKD (mean uremic concentration), as listed in Table 1. The uremic solutes were chosen according to the following criterion: *i*) classification according to the size and binding properties [33]; *ii*) highest uremic concentration/normal concentration ratio [33]; *iii*) knowing antioxidant capacity of some uremic solutes [14,34,35].

**Table 1:** **Uremic solute concentrations (mmol/L)**

	**Uremic concentration (UC)**	**Physiological concentration (PC)**
**1- L-arginine**	0.230	0.140
**2- Creatinine**	0.880	0.120
**3-** ***p*** **-cresol**	0.280	0.021
**4- Hippuric acid**	1.700	0.028
**5- Methylguanidine**	0.091	0.006
**6- Phenol**	0.110	0.015
**7- L-tyrosine**	0.110	0.027
**8- Urea**	33.000	6.700
**9- Uric acid**	0.600	0.420

**1 -** UC [59], PC [60]; **2 -** UC [61], PC [62]; **3 -** UC [63], PC [64]; **4 -** UC [61], PC [65]; **5 -** UC [66], PC [66]; **6 -** UC [67], PC [65]; **7-** UC [68], PC [69]; **8 -** UC [70], PC [62]; **9 -** UC [61], PC [64].

### ABTS^•+^ radical scavenging by uremic solutes

**Figure 1:**
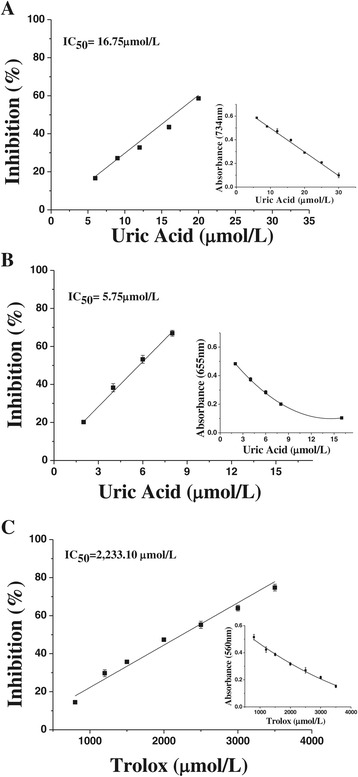
Scavenging capacity of the uric acid and Trolox on the ROS. (**A**) ABTS^•+^ scavenging capacity (IC_50_) of uric acid. The graph inset shows the mean absorbance of ABTS^•+^, at 734 nm, for various concentrations of uric acid. (**B**) HOCl/OCl^−^ scavenging capacity (IC_50_) of uric acid. The inset shows the mean absorbance of HOCl/OCl^−^, at 655 nm, for various concentrations of uric acid. (**C**) O_2_^•-^ scavenging capacity (IC_50_) of Trolox. The inset shows the mean absorbance of O_2_^•-^, at 560 nm, for various concentrations of Trolox. The scavenging capacities against oxidizing species were performed in triplicate tests.

In the ABTS^•+^ assay, the Trolox (standard) showed an IC_50_ value of 16.45 ± 0.30 μmol/L. Uric acid, with an IC_50_ of 16.75 ± 0.14 μmol/L (Figure 1A) for ABTS^•+^, was as effective an antioxidant as Trolox.

The other solutes (Table 2) also show efficiency against ABTS^•+^, with IC_50_ ranging from 4 μmol/L to 61 mmol/L or higher: *p*-cresol < L-tyrosine < phenol < uric acid < creatinine < L-arginine. Hippuric acid, methylguanidine and urea did not have any effect on ABTS^•+^ at the tested concentrations.

**Table 2:** **ROS scavenging activities of uremic solutes, expressed as concentration needed for 50% inhibition (IC**_**50**_ ± SEM*, μ**mol/L)**

**Solutes**	**ABTS** ^**•+**^	**O**_**2**_^**•-**^	**H**_**2**_ **O**_**2**_ ***	**HOCl/OCl** ^**−**^	**Crocin bleaching assay**
**L-arginine**	61,350.00 ± 562.56	**	**	92,100.00 ± 0.10	**
**Creatinine**	536.26 ± 3.32	**	**	9,130.00 ± 0.10	**
***p*** **-cresol**	3.99 ± 0.01	**	**	15.75 ± 0.12	1,162.31
**Hippuric acid**	**	**	**	1,600.00 ± 0.01	**
**Methylguanidine**	**	**	**	**	**
**Phenol**	12.98 ± 0.09	**	**	8.95 ± 0.10	1,125.81
**Trolox**	16.45 ± 0.30	2,223.10 ± 0.17	**	8.65 ± 0.27	10.09
**L-tyrosine**	5.23 ± 0.02	**	**	2.83 ± 0.04	**
**Urea**	**	**	**	5,600.00 ± 0.20	**
**Uric acid**	16.75 ± 0.14	**	**	5.75 ± 0.13	6.90

Values in μmol/L of IC_50_ of solutes; * SEM (standard error of the mean); ** No effect at the concentration used; *** Method was validated by reaction of H_2_O_2_ with catalase.

### HOCl/OCl^−^ scavenging by uremic solutes

In the HOCl/OCl^−^ assay, most of the uremic solutes proved to be effective in scavenging this ROS and, therefore, it was possible to measure their IC_50_ (Figure 1B). However, methylguanidine, even at up to 10 times the average uremic plasma level, had no effect on HOCl/OCl^−^. Trolox was used as the standard, with an IC_50_ = 8.65 ± 0.39 μmol/L. Table 2 presents the values of IC_50_ for uremic solutes in this assay, from approximately 3 μmol/L up to 92 mmol/L or higher; the ascending order of IC_50_ was: L-tyrosine < uric acid < phenol < *p*-cresol < hippuric acid < urea < creatinine < L-arginine (descending order of effectiveness).

### O_2_^•-^ scavenging by uremic solutes

The uremic solutes were tested at three concentrations: physiological, uremic and 10 times the average uremic level. None of the tested solutes were effective in scavenging O_2_^•-^ at any tested concentration. Again, Trolox was used as a standard, showing a relatively high value of IC_50_ (≈2 mmol/L), but demonstrating that the test was valid (Figure 1C).

### H_2_O_2_ scavenging by uremic solutes

None of the uremic solutes at physiological, or up to 10 times uremic concentrations, or even Trolox (up to 6 mmol/L), captured H_2_O_2_ (data not shown), so we used catalase to validate the test, because of its recognized efficiency and specificity in converting H_2_O_2_ to water and oxygen [21]; for this enzyme, an IC_50_ of 0.55 units/mL or 18.6 μg/mL (77.5 nmol/L, considering the molecular weight, 240,000 Da [36]) was observed.

### ROO^•^ scavenging by uremic solutes

**Figure 2:**
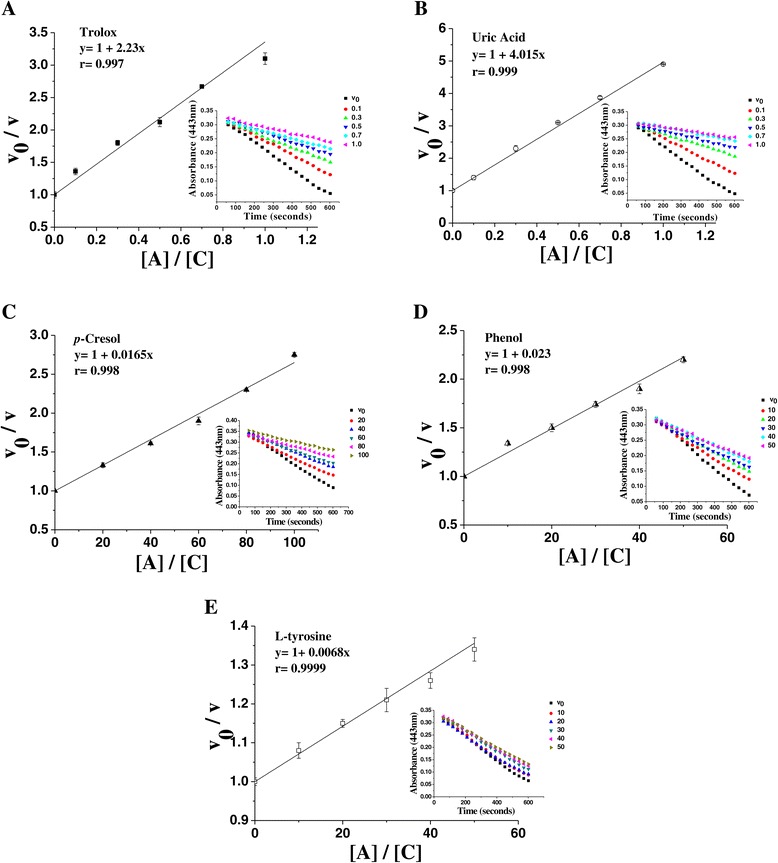
Reaction velocity ratios plotted against uremic solute (sample) concentrations in the crocin bleaching assay. (**A**) Trolox; (**B**) uric acid; (**C**) *p*-cresol; (**D**) phenol; (**E**) L-tyrosine. [C], crocin concentration and [A], sample concentration. The inset shows the decrease in absorbance of crocin, at 443 nm, over 10 min: (v_0_) velocity in the absence and (v) velocity in the presence of various concentrations of samples. The experimental conditions are described in Methods. The ROO^•^ scavenging assay was performed in duplicate testes.

The ability of Trolox to inhibit crocin bleaching is shown in Figure 2A. Comparing the values of the slope (reaction rate) for the uremic solutes, the order of decreasing antioxidant capacity was: uric acid > *p*-cresol > phenol > L-tyrosine (e.g. see Figure 2B, C, D, E). In the presence of all the other solutes, the crocin bleaching rate was unchanged, indicating a lack of interaction with the peroxyl radical and, therefore, that these solutes did not show antioxidant capacity against ROO^•^.

It is also possible to assess crocin bleaching in another way, by determining the percent inhibition of crocin bleaching (%In) [37] and thus the IC_50_. It is observed that the IC_50_ values found for Trolox and uremic solutes follow the same order of efficiency as the values of the slopes in the competitive test (the lower the IC_50_ value, the more efficient was the sample, and the greater the slope of the reaction rate regression line, showing stronger antioxidant activity) (Table 3).

**Table 3:** **Comparison between competitive kinetic slopes and IC**_**50**_ **, for the crocin bleaching assay**

**Solutes**	Slope of regression line*	**IC**_**50**_ **(**μmol/L)**
**Uric acid**	4.015	6.90
**Trolox**	2.230	10.09
**Phenol**	0.023	1,125.81
***p*** **-cresol**	0.016	1,162.31
**L-tyrosine**	0.007	***
**Methylguanidine**	9.272x10^−4^	***
**L-arginine**	7.727x10^−4^	***
**Hippuric acid**	8.000x10^−5^	***
**Creatinine**	6.776x10^−5^	***
**Urea**	2.558x10^−6^	***

*Regression line slopes in order of decreasing efficacy; **IC_50_ in order of decreasing efficacy, from plot of equation: % In = (1- (Δv/Δv_o_)) × 100; ***IC_50_> highest concentration tested.

It can be seen that uric acid, Trolox, phenol and *p*-cresol exhibited significant antioxidant effects, while the other solutes did not. It should be noted that only uric acid produced an effect of the same order of magnitude as Trolox, which shows its importance for the model system of LPO, since its IC_50_ was lower than the physiological concentration and its activity was approximately twice that of Trolox (Table 3).

### Oxidant scavenging of uremic solute mixtures

Considering the four solutes: uric acid, *p*-cresol, phenol and L-tyrosine that exhibited significant scavenging capacity towards the three reactive species ABTS^•+^, HOCl/OCl^−^ and ROO^•^, we performed the assays with mixtures. Therefore, the antioxidant behaviors of the mixtures of these solutes were investigated, using the IC_50_ of each solute against each ROS as a reference.

**Figure 3:**
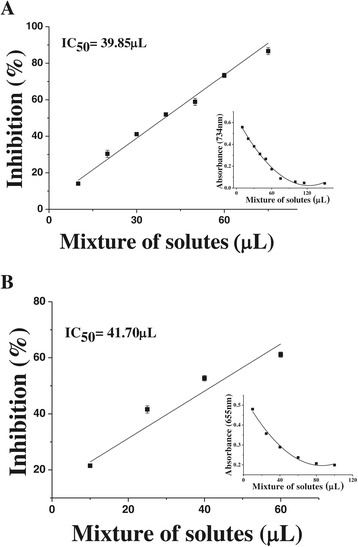
Capacity of the mixture of solutes uric acid, phenol, *p*-cresol and L-tyrosine to scavenge (A) ABTS^•+^ and (B) HOCl/OCl^−^. The inset of the figure (**A**) show the mean absorbance of ABTS^•+^, at 734 nm, for various volumes in the assay of the stock solute mixture with: uric acid (33.5 μmol/L), phenol (25.96 μmol/L), *p*-cresol (7.98 μmol/L) and L-tyrosine (10.46 μmol/L); final volume reaction, 300 μL. The inset of the figure (**B**) show the mean absorbance of HOCl/OCl^−^, at 655 nm, for various volumes in the assay of the stock solute mixture with: uric acid (57.5 μmol/L), phenol (89.5 μmol/L), *p*-cresol (157.5 μmol/L) and L-tyrosine (28.3 μmol/L); final volume reaction, 1500 μL. The scavenging capacities against oxidizing species were performed in triplicate tests.

In the ABTS^•+^ or HOCl/OCl^−^ scavenging assays for 4-solutes mixtures, it was found practically the projected volume of solute mixture needed for IC_50_ (39.85 μL and 41.70 μL, respectively) (Figure 3A and B). For both assays, the concentration of each solute is presented in Table 4A and B, which represented about a quarter of its individual IC_50_.

**Table 4:** **Relations between the IC**_**50**_ **of the solutes uric acid, phenol,** ***p*** **-cresol and L-tyrosine for their scavenging of: (A) ABTS** ^**•+**^ **, (B) HOCl/OCl** ^**−**^ **and the IC**_**50**_ **of mixed samples**

**Uremic solutes in mixture**	**Volume (μL) of solute mixture needed for IC**_**50**_	**Concentration (μmol/L) of each solute in mixture at IC**_**50**_	**IC**_**50**_ **(μmol/L) of single solutes**	**Ratio of concentration of each solute in the IC**_**50**_ **of the mixture to its respective IC**_**50**_ **, for ABTS** ^**•+**^
**A**	**ABTS** ^**•+**^	**ABTS** ^**•+**^	**ABTS** ^**•+**^	**ABTS** ^**•+**^
***p*** **-cresol**		1.06	3.99	0.26
**L-tyrosine**	39.85	1.39	5.23	0.26
**Phenol**		3.45	12.98	0.26
**Uric acid**		4.45	16.75	0.26
**B**	**HOCl/OCl** ^**−**^	**HOCl/OCl** ^**−**^	**HOCl/OCl** ^**−**^	**HOCl/OCl** ^**−**^
**L-tyrosine**		0.78	2.83	0.27
**Uric acid**	41.70	1.60	5.75	0.27
**Phenol**		2.50	8.95	0.27
***p*** **-cresol**		4.38	15.75	0.27

**Figure 4:**
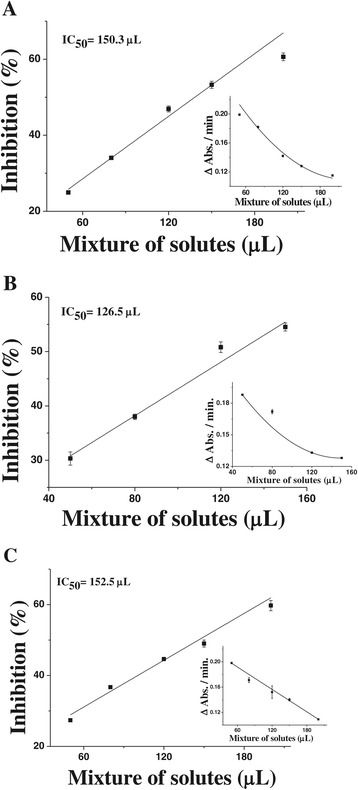
**Effects of solute mixtures in the crocin bleaching assay. (A)** uric acid plus phenol; **(B)** uric acid plus *p*-cresol and **(C)** phenol plus *p*-cresol. The insets show the mean rates of change of absorbance, at 443 nm, in crocin bleaching plotted against amount of solute mixture added: **(A)** stock solute mixture with: uric acid (69.0 μmol/L) plus phenol (11,258.1 μmol/L), **(B)** stock solute mixture with: uric acid (69.0 μmol/L) plus *p*-cresol (11,623.1 μmol/L) **(C)** stock solute mixture with: phenol (11,258.1 μmol/L) plus *p*-cresol (11,623.1 μmol/L); final volume reaction, 2000 μL. The ROO^•^ scavenging assay was performed in duplicate testes.

In the crocin bleaching assay, it was used to binary mixtures: uric acid + phenol; uric acid + *p*-cresol; phenol + *p*-cresol, then it was found a high volume of solute mixture needed for IC_50_ (150.3 μL, 126.5 μL, 152.5 μL, respectively) (Figure 4A, B, C and Table 5).

**Table 5:** **Relations between the IC**_**50**_ **of the solutes uric acid, phenol and** ***p*** **-cresol determined in the crocin bleaching assay, with single solutes or binary mixtures**

**Mixtures of uremic solutes**	**Volume (μL) of solute mixture needed for IC**_**50**_	**Concentrations (μmol/L) of each solute in mixture at IC**_**50**_			**Total concentration of uremic solutes observed in the mixture (μmol/L)**	**IC**_**50**_ **(μmol/L) of single solutes**			**Projected total concentration (μmol/L) of the binary mixtures of uremic solutes from the IC**_**50**_ of a single solutes*
		**Uric acid**	**Phenol**	***p*** **-Cresol**		**Uric acid**	**Phenol**	***p*** **-Cresol**	
**Uric acid + Phenol**	150.3	5.18	846.04	---	851.22	6.90	1125.81	---	566.35
**Uric acid +** ***p*** **-cresol**	126.5	4.36	---	735.16	739.52	6.90	---	1162.31	584.60
**Phenol** ***+ p*** **-cresol**	152.5	---	858.43	886.26	1744.69	---	1125.81	1162.31	1144.06

*The projected total concentration at the IC_50_ of the binary mixtures, for example, uric acid + phenol is (6.90 + 1125.81)/2 = 566.35.

## Discussion

In view of the quali-quantitatively wide variation of uremic solutes found in biological fluids, and the ability of some of these solutes to scavenge certain ROS, we employed a broad set of measures of antioxidant capacity, rather than a single measure, to obtain a highly representative result. These analytical techniques were chosen to reflect, as accurately as possible, the antioxidant capacity of uremic solutes. Data from this study showed that the uremic solutes *p*-cresol, phenol, L-tyrosine and uric acid were effective antioxidants, mainly through three model systems: ABTS^•+^, HOCl/OCl^−^ and crocin bleaching assay (Table 2). When mixtures of these solutes where studied, it was found that, in 4-solutes mixtures, each one of the solute captured 12.5% for the IC_50_ in ABTS^•+^ or HOCl/OCl^−^ assays (Table 4A and B), exhibiting a virtually exact additive effect; in 2-solutes mixtures for ROO^•^ capture, it was observed the need of more mass of uremic solutes to reach an IC_50_ value of mixtures, which was higher than the projected IC_50_ obtained from the IC_50_ of single solutes (25% of each, in the binary mixtures) (Table 5).

In the ABTS^•+^ assay, Trolox was used as “standard” and showed an IC_50_ value similar that found with uric acid (Figure 1A), as also noted by others [38,39]. In a study of Gülçin [40], 79.90 μmol/L Trolox and 110.37 μmol/L L-tyrosine both proved to be effective, scavenging 95% and 62% of ABTS^•+^, respectively. The presence of hydroxyl groups and aromatic rings in the uremic solutes appears to be correlated with their antioxidant activity, since the most effective solutes were *p*-cresol, L-tyrosine, phenol and uric acid (Table 2); this relationship between structure and antioxidant capacity of compounds has also been reported by others [40,41].

Mayer et al. [16] investigated the effect of HD on the antioxidant capacity of serum patients using the ABTS^•+^ assay. The authors observed a decrease in serum total antioxidant capacity during the HD treatment, which was attributed to the dialytic removal of uric acid and ascorbic acid. Similarly, Bianchi et al. [42] investigated the consequences of an HD session on the systemic oxidative stress of CKD patients, assaying serum samples with the total reactive antioxidant potential (TRAP) test; when pre- and post-HD sera of CKD patients were compared, it was observed a reduction in TRAP after one HD session.

Most of the uremic solutes proved to be effective in capture HOCl/OCl^−^, with exception of methylguanidine, which showed no effect (Table 2). The relationship of uremic solutes with HOCl/OCl^−^ system seems to be relevant when we consider the data about the increase in both the activity and concentration of myeloperoxidase (MPO) in HD patients when compared with pre-HD and control subjects [43,44]. Increased MPO activity in HD patients may be due to the activation of neutrophils during the forced blood passage through the dialysis circuits, which is related to the precocity and prevalence of atherosclerotic disease in CKD patients. In fact, increased levels of proteins modified by HOCl generated by the MPO/H_2_O_2_/Cl^−^ system have been found in serum of HD patients, such as oxidized albumin; HOCl-modified albumin impairs the association of high-density lipoprotein (HDL) with the scavenger receptor class B type I, which have a protective role against atherosclerotic cardiovascular disease [45]; also, it was found a direct relationship between plasma levels of oxidized low density lipoprotein (ox-LDL) and MPO in CKD diabetic patients during HD [46]. On the other hand, whereas most of the uremic solutes scavenged ABTS^•+^ and HOCl/OCl^−^ effectively, the same did not happen with the other biological ROS, O_2_^•-^ and H_2_O_2_ (Table 2).

O_2_^•-^ is an essential ROS, since it is a precursor for other reactive species and it is constantly produced in the human body during physiological processes, such as the mitochondrial electron transport chain and some signaling events in the vascular system [21]. Its production is also initiated in activated phagocytes into the oxidative burst, leading to the synthesis of O_2_^•-^ and other ROS, overcoming the local antioxidant capacity [21,47]. Thus, increased O_2_^•-^ levels can, for example, contribute greatly to the endothelial dysfunction and thus hinder the process of vascular wall distention by scavenging NO to generate peroxynitrite [7,21]. In this context, the main mechanism by which L-arginine leads to beneficial effects on CKD has been attributed to an increase in the NO production, since nitric oxide synthase (NOS) activity in kidney failure is determined by L-arginine concentration [48]; so, L-arginine in CKD is important to oppose endothelial dysfunction. In a review by Baylis [49], it is pointed that the total NO production is decreased in CKD patients due to main possible causes: *i*) the limitation of substrate (L-arginine) for NOS, and *ii*) the increased levels of circulating endogenous inhibitors of NOS, particularly asymmetric dimethylarginine.

According our data, none of the tested solutes were effective in scavenging O_2_^•-^ at any tested concentration (Table 2). Our results corroborate those of Barreiros et al. [50], which reported that uric acid was inert against O_2_^•-^ and H_2_O_2_, but showed strong reactivity with ROO^•^ and NO_2_^•^. In view of the high activity against ROO^•^, it has been postulated that uric acid protects lipids and DNA from the interaction with ROO^•^. While our findings of O_2_^•-^ scavenging indicated that Trolox has a high IC_50_, (Figure 1C), Ak and Gülçin [51] and Gülçin [40] reported a greater efficiency of Trolox against O_2_^•-^, when they used a more sensitive chemiluminescent method, with inhibition of 78.2% of O_2_^•-^ activity at 39.95 μmol/L, indicating that these differences may be attributed to methodological sensitivity.

Miyamoto et al. [35] showed that the uremic solutes uric acid, *p*-cresol and indoxyl sulfate, under physiological concentrations, they have potent antioxidant activity, comparable to that of superoxide dismutase, assessed by luminol chemiluminescence, which represents the ability to scavenge O_2_^•-^. In this study, O_2_^•-^ was generated from the xanthine/xanthine oxidase system, and the authors found that, at least for indoxyl sulfate, it did not inhibit the xanthine oxidase activity, confirming that the antioxidant property of this solute is exclusively due to its capacity to scavenge O_2_^•-^.

H_2_O_2_ is produced *in vivo* continuously in virtually all tissues. The mitochondria can contribute with the cellular generation of H_2_O_2,_ by both monoamine oxidase activity and dismutation of O_2_^•-^ generated in the electron transport chain, although mitochondria can also consume H_2_O_2_; thus, in *in vivo* systems, H_2_O_2_ is generated by O_2_^•-^ dismutation, spontaneously or catalyzed by superoxide dismutase, as well as by β-oxidation of fatty acids or directly by various oxidase enzymes [21]. None of the uremic solutes or even Trolox at high levels captured H_2_O_2_ (data not shown) (Table 2).

Lipid bilayer cell membranes are the main targets of attack by free radicals, causing loss of membrane structure and functionality; therefore, LPO is a part of the etiology of many diseases. Such serious consequences of LPO have encouraged studies on the efficacy and mechanisms of action of biological antioxidants, justifying the relevance in the understanding the activity of uremic solutes against ROO^•^. In the crocin bleaching assay, antioxidants compete with crocin for the ROO^•^ radical generated by AAPH thermolysis; therefore, the inhibition of the oxidation of crocin depends on the capacity of the samples to capture this radical species generated *in situ*. The solutes uric acid, *p*-cresol, phenol and L-tyrosine, and Trolox, were effective in inhibiting the crocin bleaching (Figure 2A, B and C; Tables 2 and 3); it should be noted that only uric acid produced an effect of the same order of magnitude as Trolox. It is interesting to note that the increased circulating levels of uric acid has been cited as a protective mechanism trying to counteract LPO oxidation during atherosclerosis, although this increase has been also associated with an elevated rate of disease and mortality [34].

The relationship between the antioxidant potential of uremic solutes and the ability to prevent biological damage was also assessed by Ujhelyi et al. [14]; the authors showed that plasma ultrafiltrate from CKD patients exhibited *i*) a pronounced antioxidant activity, assessed by the ability to inhibit the heme-mediated LDL oxidation (*in vitro* assay), and *ii*) protection against endothelial cytotoxicity induced by LDL oxidation. This antioxidant capacity of plasma ultrafiltrate from CKD patients was lost after HD as a consequence of the dialytic removal of some compounds, including the uremic solutes indoxyl sulfate, *p*-cresol, phenol, and uric acid; in addition, it was observed that the retention of other solutes, including L-arginine, creatinine, guanidines, hippuric acid, among others, was not sufficient to prevent the oxidative modification of LDL. The presence or absence of antioxidant ability observed by the authors was corroborated by our findings for the most of the uremic solutes. At this time, it is appropriate to quote a sentence of Ujhelyi et al. [52] in response to Meijers and colleagues, “*p-cresol can be considered to be a Janus-faced compound with several toxic and some beneficial properties, and it might be a marker of pathologic metabolic processes that lead to the observed enhanced risk of mortality in hemodialysis patients*”. This Janus-faced feature has been also attributed to other solutes, such as uric acid [53] and indoxyl sulfate [35,54].

Bianchi et al. [42] assessed the oxidative damage of lipids in erythrocytes of CKD patients by chemiluminescence assays for LPO initiated by *tert*-butyl hydroperoxide (t-BOOH), which are sensitive and have been used to observe indirectly the previous oxidative stress and diminished endogenous antioxidants [55,56]. When chemiluminescence data from pre- and post-HD patients were compared, no differences were observed, but an increased chemiluminescence was observed when pre-HD patients were compared with healthy subjects, showing a state of oxidative stress in these patients. It is important to note that the crocin bleaching test allows the analysis of the antioxidant effect of uremic solutes, i.e., how much they prevent the LPO process.

Recently, an interesting approach to evaluate the relationship between uremic solutes and oxidative stress was proposed by Oowada et al. [19]: the authors used a common experimental procedure to produce ROS (UV/visible-light photolysis of free radical precursors/sensitizers), and they made a radar chart based on the recording of the electron spin resonance spectrum using spin traps; this approach was used to assess the scavenging capacity of serum samples. The authors observed that serum samples of CKD patients showed decreased scavenging capacity against ^•^OH and methyl (^•^CH_3_) radicals and singlet oxygen (^1^O_2_), increases for O_2_^•-^ and RO^•^ radicals, and no changes for ROO^•^ radical in comparison to healthy individuals. It is interesting to note that the authors did not explain these changes in the scavenging capacity, but they highlight for the future application of this approach as a tool for clinical uses.

Given that in the body and especially under uremic conditions, there is always a complex mixture of uremic solutes, it would clearly be of interest to analyze the effects of a mixture of the four solutes, uric acid, *p*-cresol, phenol and L-tyrosine, that exhibited significant scavenging capacity towards the three reactive species ABTS^•+^, HOCl/OCl^−^ and ROO^•^. In the ABTS^•+^ and HOCl/OCl^−^ scavenging assays carried out with the 4-solutes mixture, it was found that at the IC_50_, the concentration of each solute was about a quarter of its individual IC_50_ (Table 4A and B)_._

In view of the complexity of the crocin bleaching assay, in which antioxidants compete dynamically with the crocin to react with the radical species generated *in situ,* the mixed-solute in this assay was restricted to binary mixtures. To scavenge ROO^•^, it was need more mass of uremic solutes to reach an IC_50_ value of these mixtures, which was therefore higher than the projected IC_50_, obtained from the of the IC_50_ of single solutes (25% of each, in the binary mixtures) for the same assay (Table 5). Similarly, using the ORAC (oxygen radical absorbance capacity) method, Noguer et al. [57] observed that binary mixtures of uric acid or ascorbic acid with the phenolic compound 3-hydroxyphenylacetic acid showed a diminished antioxidant activity in comparison with the theoretical values obtained from the sum of the activity of each compound individually. Taken together, it can be suggested the existence of an interaction between phenol and/or phenolic derivatives with uric acid or even with other antioxidant compounds. In front of these findings, it seems extremely useful the monitoring the effectiveness of the antioxidant status of biological samples (mixtures), mainly under conditions of altered levels of the endogenous antioxidant network and/or after the employment of exogenous antioxidant therapy.

Considering our findings, the IC_50_ showed to be a valuable analytical tool, as knowing the values of IC_50_ for each solute, it was possible to weigh their ROS-scavenging capacities in mixtures of solutes. This analysis was accurate and exact for the assays in which the ROS were present in the initial mixture (ABTS^•+^, HOCl/OCl^−^), but was not exact for those in which they were generated *in situ* (crocin bleaching assay, ROO^•^). Thus, the application of the IC_50_ as an analytical tool to mixtures of solutes enables their individual antioxidant activities to be investigated in biological fluids containing such mixtures, over a range of concentrations and in varied pathophysiological states with or without antioxidant therapy, such as those provoked by kidney disease.

## Conclusions

From the results of this study, we can conclude that the same set of uremic solutes, uric acid, *p*-cresol, phenol and L-tyrosine, showed significant antioxidant activity in the ABTS^•+^, HOCl/OCl^−^ and crocin bleaching (ROO^•^ capture) assays, albeit with differing efficiencies. The values of IC_50_ found, in the three assays where the set of four uremic solutes were effective, they were below physiological concentrations, as demonstrated by their behavior both separately or combined. It must be emphasized, however, that for O_2_^•-^ and H_2_O_2_, which are extremely important endogenous ROS produced during oxidative stress, no uremic solute showed scavenging capacity.

Thus, it could be interesting to knowledge the behavior (IC_50_) of theoretical mixtures and compare them with real biological samples using, for example, an analytical approach to generate ROS and to monitor their specific scavenging capacities, as the model proposed by Oowada and collaborators [19]. Finally, the combined use of these analytical tools may be useful for the assessment of the oxidative status of CKD and HD patients, as well as to project and to monitor the effectiveness of antioxidant interventions.
